# Postmenopausal women's experiences of a resistance training intervention against vasomotor symptoms: a qualitative study

**DOI:** 10.1186/s12905-022-01900-0

**Published:** 2022-07-30

**Authors:** Emilia Berin, Anna-Clara Spetz Holm, Mats Hammar, Lotta Lindh-Åstrand, Carina Berterö

**Affiliations:** 1 Department of Obstetrics and Gynaecology in Linköping, and Department of Biomedical and Clinical Sciences, Linköping University, 581 85 Linköping, Sweden; 2 Division of Nursing Sciences and Reproductive Health, Department of Health, Medicine and Caring Sciences, Linköping University, Linköping, Sweden

**Keywords:** Menopause, Resistance training, Strength training, Qualitative research, Vasomotor symptoms, Hot flushes, Exercise motivation

## Abstract

**Introduction:**

Resistance training may be an effective intervention to improve menopausal symptoms and increase women’s quality of life. However, most postmenopausal women do not perform regular resistance training. The purpose of this study was to explore postmenopausal women’s experiences of participation in a resistance-training intervention to find barriers and motivators for the training.

**Methods:**

Fifteen postmenopausal women with low physical activity, who participated in a randomized controlled trial evaluating the effect of a resistance-training program on vasomotor symptoms and health-related outcomes, were consecutively recruited to this qualitative study. After completion of the 15-week resistance-training program, they took part in individual semi-structured interviews, followed by a telephone interview 1 year later. All interviews were transcribed verbatim and thematic analysis was used to analyse the data.

**Results:**

The analysis generated three themes that were involved at different time points. These were: “Trigger—Hopes of symptom relief”, “An evolving motivation as a driving force for change” and “Finding new triggers”*.* Accountability, and continuous professional and emotional support, were factors that fueled the women’s motivation to perform regular resistance training during the study. Resistance training improved general well-being and most women experienced improvement in vasomotor symptoms. The women’s motivation changed from being driven by a wish to improve bothersome symptoms, into a wish to achieve feelings of well-being and enjoyment. The change was seen regardless of effects of the intervention on vasomotor symptoms.

**Conclusion:**

This first qualitative evaluation of physical exercise as an intervention to treat vasomotor symptoms in postmenopausal women, found that the symptoms acted as a motivational trigger to initiate resistance training in low-active women. The motivation to exercise changed during the intervention from a wish to ameliorate symptoms into something the women did for enjoyment and well-being in general. This change in motivating factors may have contributed to a behavior change since all participants had increased their physical activity after 1 year regardless of effects on VMS.

*Trial registration* The trial was preregistered at ClinicalTrials.gov; www.clinicaltrials.gov, ID: NCT01987778, date of first registration: 19/11/2013.

**Supplementary Information:**

The online version contains supplementary material available at 10.1186/s12905-022-01900-0.

## Introduction

Menopause is the final menstrual bleeding and can be diagnosed after 12 months of amenorrhea. Menopause marks the end of a woman’s reproductive life and affects all women who live long enough. It occurs when the decreasing oestrogen production from the ovaries becomes too low to stimulate endometrial growth. The age of natural menopause differs between individuals but generally occurs between 45 and 57 years. Although a physiologic event, menopause is often accompanied by symptoms that may have a large impact on women’s daily life and decrease quality of life substantially. Hot flushes and sweating (vasomotor symptoms, VMS) are the hallmark symptoms of the menopausal transition, reported by around 75% of all women [1]. The VMS can be described as an intense sensation of heat in the face, neck and chest, sometimes accompanied by sweating followed by shivering. VMS may disrupt sleep, affect mood, work, concentration, and social activities and every fourth woman reports a need for treatment [2, 3].

Other common symptoms reported around menopause include sleep disturbances, mood changes, anxiety, depressive symptoms, cognitive changes, and joint pain [4, 5]. Contrary to VMS, those symptoms are not as clearly associated with the hormonal changes of menopause as the VMS but are probably more related to general aging and influenced by somatic, psychologic, and social factors [4]. Nevertheless, they contribute to a decreased quality of life during the menopausal transition and beyond.

Of importance to women’s health, the hormonal changes that occur during the menopausal transition also cause unfavourable metabolic changes. Fat distribution shifts towards abdominal fat accumulation, which is associated with metabolic syndrome, type 2 diabetes, and an increased risk of cardiovascular disease after menopause [6]. In addition, the physical and psychological symptoms of menopause often influence each other; for example, VMS often disturb sleep, which may affect mood and in turn reduce the ability to cope with VMS.

The most effective treatment for VMS, that also improves quality of life in symptomatic women, is oestrogen-containing menopausal hormone therapy (MHT) [7, 8]. However, MHT is not available to all women due to contraindications and many women are reluctant to use MHT because of fear of adverse effects. Consequently, there is a need to find effective and safe non-hormonal treatments for VMS to reduce symptoms and improve quality of life.

One non-hormonal treatment that has been suggested is physical exercise. Observational studies have found associations between regular exercise and fewer/milder VMS [9]. Moreover, a higher body mass index and increasing body fat has been associated with more frequent and severe VMS in longitudinal studies [10]. Exercise is an appealing intervention because it may counteract many of the deleterious effects on physical and mental health that are associated with aging and menopause. Women who exercise regularly have a lower risk of cardiovascular disease, type 2 diabetes, dementia and depression [11]. In addition, exercise may improve sleep, mood, decrease anxiety, decrease pain, and improve overall quality of life.

Evidence from intervention trials about the effect of exercise on VMS is insufficient, partly since previous trials have had problems with low compliance or high drop-out rates [12]. To try to fill the evidence gap, we performed a randomized controlled trial (RCT) of resistance training as a treatment for VMS in postmenopausal women. We found that VMS decreased by almost 50% among women randomized to 15 weeks of resistance training compared to a control group, and that menopause-specific quality of life improved in the resistance-training group [13, 14]. The included women had low physical activity levels before the study, and the intervention meant a significant change in lifestyle for the women if they were to perform it as intended.

Naturally, adherence to a treatment is crucial to achieve benefits, and adherence in the long term is especially important with exercise to achieve positive health effects. Since previous intervention trials in postmenopausal women have been affected by attrition or low compliance, there is a need to better understand what affects uptake and adherence to an exercise intervention in this patient group [12, 15]. To our knowledge, none of the previous intervention trials of exercise for VMS included a qualitative evaluation. With qualitative methodology it is possible to attain a deeper insight into the perspectives and experiences of the participating women, which in addition may enrich and complement the results obtained from quantitative analysis. We therefore performed a qualitative study with the aim to explore postmenopausal women’s experiences of participation in a resistance-training intervention.

## Methods

### Context

This qualitative study was part of a larger randomized controlled trial with the primary aim to investigate the effect of a resistance-training program on vasomotor symptoms in postmenopausal women. The context of the RCT is described below.

### Intervention trial

In the original RCT, 65 postmenopausal women were randomized to either a 15-week resistance-training program or a non-treated control group. Recruitment took place by means of advertisements in local newspapers and posters displayed at the University Hospital of Linköping, Sweden. Prior to inclusion, all participants received written and oral information about the study, including this qualitative study, and gave their written informed consent to take part. The study was approved by the Regional Ethical Review Board in Linköping (2013/285-31).

#### Inclusion criteria

The inclusion criteria in the RCT were as follows: postmenopausal women, ≥ 45 years old, ≥ 4 moderate-severe vasomotor symptoms/24 h, low-active (maximum 225 min physical activity per week of any intensity of which a maximum of 75 min of vigorous intensity), not on medication for vasomotor symptoms (including natural remedies), blood pressure < 160/100, capillary haemoglobin > 110 g/l, and being physically able to perform resistance training three times/week (Additional file 1).

#### Intervention

The 15-week resistance-training program was designed to activate all major muscle groups and result in improved muscle strength and hypertrophy. It was performed three times/week with one session/week in the presence of a physiotherapist. The physiotherapist followed up on their progress and adjusted/increased loads. The other sessions they exercised independently during the gym’s opening hours. Attendance was logged via the gym’s electronic card system and in a personal logbook at the gym. The mean attendance was 2.2 training sessions per week. The gym was a public fitness centre within walking distance from the city centre and easily accessible by public transportation or car. Members include both sexes and a variety of ages, and the gym offers group training classes as well as a resistance-training area.

### Data collection

Participants in the intervention group were invited to individual interviews following completion of the 15-week resistance-training program. They were consecutively recruited regardless of adherence to the intervention and interviewed in a meeting room at the university. The setting was distanced from the clinic where the visits in the study took place, in a different building at the University Hospital area. The interviews were semi-structured, using an interview guide with four questions to assist the interviewer and ensure that relevant topics were covered during the interview. The semi-structured interview allows for new questions to emerge during the dialogue depending on the information shared by the participant, and facilitates the collection of data of importance to the research question [16].

The interviews started with a question about menopausal symptoms (“Please, tell me about the climacteric symptoms you experienced”), then concerned the decision to join the resistance-training study and lastly focused on the experience of a possible lifestyle change during the study (“What made you join this project?”, “Is there anything in the project that has made you change your lifestyle/habits? How?”, “Is there anything that has been an obstacle for you?”). Probes and follow-up questions were used during the interviews to increase the depth of responses and generate richer descriptions. All interviews were audio-recorded and transcribed verbatim. Information on demographics, history of menopausal symptoms and previous treatments were collected in conjunction with the interview.

A follow-up telephone interview was conducted with all participants 1 year after the first interview with the purpose to follow up on adherence to the resistance training. The telephone interviews were shorter and started with a presentation of the purpose to follow up on the first interview regarding the resistance-training study. They started with a question about present climacteric symptoms (“Could you please tell me about your climacteric symptoms at this time?”) and follow-up questions about exercise, adherence to resistance training and a possible change in lifestyle were asked if the participant did not mention it spontaneously (“Do you perform any exercise?”, “What kind?/To what extent?/How often?”, “Do you perform resistance training?”, “Is there anything in the project that has made you change your lifestyle/habits?”, “Is there anything that has been an obstacle for you?”).

From the 29 women who completed the resistance-training program in the intervention study, 15 were included in this qualitative study. The decision to finish recruitment for this qualitative study was made when all eligible women until the third round of inclusion in the original RCT had been interviewed in this study. The collected data thus far was regarded as rich and meaningful, the sample was varied and no obvious new data was generated from the last interviews [17, 18]. The mean duration of the first interviews was 19 min (range 9–30) and the telephone interviews lasted for a mean of 6.5 min (range 5–10).

The mean age of the participants was 56.6 years (range 49–68) and they had experienced VMS for 1 to 18 years. Sociodemographic characteristics are presented in Table 1. Twelve of the 15 interviewed women considered their symptoms had improved during the trial.

**Table 1:** Sociodemographic characteristics of participants

**Age, years**	Mean (range)
	56.6 (49–68)
**Age, years, categorical**	N
49–50	3
51–55	5
56–60	2
61–65	4
65–68	1
**Time since menopause, years**	Mean (range)
	5.7 (1.1–16.25)
	Median
	4.1
**Duration of vasomotor symptoms, years**	Mean (range)
	5.9 (1–18)
**Social status**	
Married or living with partner, N	13
Single, living alone, N	2
Living with children, N	6
Single parent, N	2
**Employment status**	
Employed, N	13
Retired, N	2

The table shows sociodemographic characteristics of included participants. N = 15

### Data analysis

**Fig. 1:**
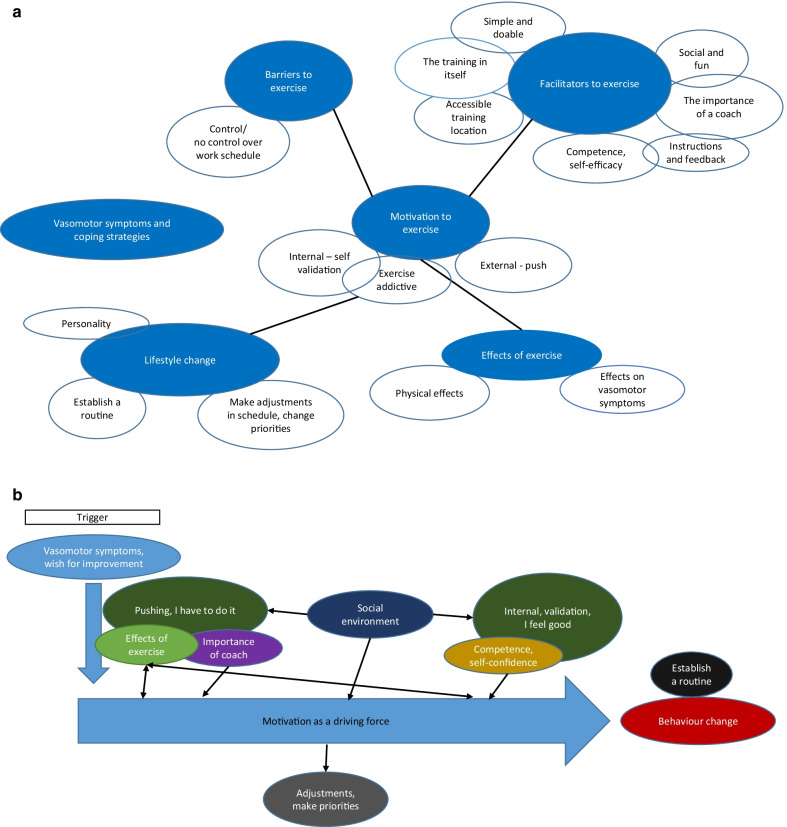
**a** Preliminary thematic map showing preliminary themes and subthemes, **b** reworked thematic map after revision of themes. Themes are close to final

**Fig. 2:**
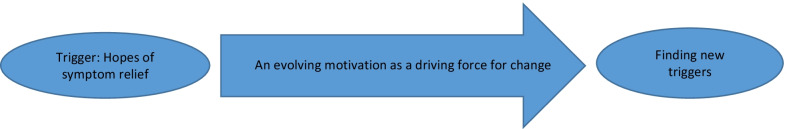
The final three themes that were constructed as a result of the analysis. The map shows the themes as a process

The data were analysed using thematic analysis as described by Braun and Clarke [19]. A thematic analysis was chosen since it provides a flexible method to identify and interpret patterns in data. Each interview transcript was thoroughly read and searched for initial codes that were noted. The transcripts were thereafter coded systematically, and the codes sorted and grouped into potential themes, resulting in a thematic map being constructed for each interview. The researchers first coded the transcripts independently and then compared and discussed the codes until consensus was reached. A thematic map was constructed for each interview to visualize the themes, subthemes, and relationships between them. The codes and initial thematic maps were constructed by EB and CB independently and then compared and discussed, generating a comprehensive thematic map with six themes and several subthemes (Fig. 1a, b). The themes were then refined in an analytic process that included reading the transcripts several times, and further discussing the content of each theme between the researchers. This resulted in identification of three themes (Fig. 2). To verify the themes in relation to the data, all transcripts were read again as a whole. See Table 2 for a description of the analytic phases.

**Table 2:** Phases of data analysis

Phase	Description	Example	
1. Data familiarisation	Transcripts from interviews were read several times and initial notes and thoughts were written down	“Positive experience, no obstacles” (notes Participant 6)	
2. Generation of initial codes	Coding was performed separately by two researchers (EB, CB), and data extracts collated within each code. Codes were then compared and discussed between the two researchers	”I have a craving for it (the strength training) now, because I think it’s so fun, so I still exercise at least three times per week”	Initial code: Developed craving for strength training
3. Generation of initial themes	Codes were collated into initial themes and subthemes independently by two researchers (EB, CB), a thematic map was constructed for each interview	Initial code: “Developed craving for strength training”	Initial theme: Lifestyle change—exercise addictive
4. Initial themes reviewed	The initial themes were reviewed by reading all data extracts belonging to each initial theme and the initial themes reworked to generate a preliminary thematic map of the whole data set. The themes were further reviewed by re-reading the whole data set and ensuring the accurate representation of the raw data in the themes	See Fig. 1a and 1b for example of the preliminary thematic map, and reworked thematic map	
5. Refining and defining themes	The themes were refined in an analytic process of discussing the meaning and definition of each theme, and checking the final themes against the raw data set	See Fig. 2 for final thematic map	
6. Report writing	The themes presented in a report with definitions of each theme and the story they tell, along with data extracts represent the themes		

The table shows the phases of thematic analysis that were undertaken. Steps 4 and 5 involved the whole research group on several occasions during the process

## Results

The analysis revealed a process with a starting point, a “trigger” for taking part in the intervention. The themes that were identified from the interviews were the trigger “Hopes of symptom relief”, the continuing process “An evolving motivation as a driving force for change*”* and “Finding new triggers” (Fig. 2). Several factors that gave input to the process are described and defined below.

### Trigger: hopes of symptom relief

The trigger that caused the participants to apply for the intervention trial was the possibility that it would relieve their climacteric symptoms with hot flushes and night sweats (vasomotor symptoms). The vasomotor symptoms were described as the classical symptoms including feelings of warmth in the face, neck, and chest, sometimes with flushing and mostly accompanied by sweating. The symptoms had a large impact on the daily lives of the women by disrupting work and activities, limiting choices of clothes, and causing social awkwardness. Many had night sweats that affected their sleep quality. Some also described being emotionally sensitive, or in a low mood. Most participants had tried different treatments for VMS before the study, like oestrogen therapy, natural remedies, or yoga. Now they were either unable (due to contraindications) or unwilling to use oestrogen and had not experienced symptom relief from previous treatments. Some participants had exercised regularly in the past and viewed partaking as an opportunity to start regular exercise again. The hopes of relief from climacteric symptoms hence triggered the participants to apply for the study.

### An evolving motivation as a driving force for change

During the intervention, the participants’ motivation was found to be the key factor that drove them to adhere to the resistance training and make changes in their life to make the training possible. The motivation itself was a process that changed over the course of the intervention trial. It was initially triggered by the hopes of symptom relief, but during the intervention, motivation evolved through influence from different kinds of input that changed during the intervention.

Initially, the trial was a push to start exercising. The women felt accountable to the researchers and did not want to let them down or be a disappointment. It was considered important to do the exercise since the women had signed up for it when they joined the trial, and to not follow through with the program meant that they would let both themselves and the researchers down. They felt lucky to have been randomized to the intervention group and therefore felt obligated to follow through with the program. In addition, it was considered a special opportunity to have a physiotherapist who followed their progress weekly, and the participants did not want to waste the opportunity by not performing the training.

*I think I managed seven minutes on one of these Cross-trainers, and just felt that no, this is, if I get a chance like this, get an opportunity like this. To get rid of my symptoms, but at the same time increase my fitness, I must give it all I have. I can’t join this half-heartedly; it must be whole-heartedly. (Participant 11)*

The women also felt accountable to the physiotherapist. To know that someone checked on them and had expectations on them motivated them to go to the gym even when they were tired or did not feel like it. If they had missed a training session, the physiotherapist would notice it in their training log, even though only one session per week was scheduled with the physiotherapist. Expectations from family members, colleagues and friends affected the participants as well. Many had talked about the intervention trial with their relatives and colleagues, which created pressure to keep up the regular resistance training. They “had to do it” since they had told the people around them that they would.

*I have talked a lot about this, with others, shared, so there are many who… well, then I couldn’t disappoint, I couldn’t stop since I had talked about this with so many and told everyone that this, this has helped me. No, I have never thought about quitting (Participant 10)*

For some participants, the test of muscle strength that awaited at the end of the intervention functioned as an incentive to adhere to the resistance training. If they did not increase their muscle strength, it would be apparent that they had not performed the exercise.

During the trial, secondary goals also motivated some of the women to exercise; they wished to get fit, lose weight or become leaner. These goals became important for some of the participants to convince themselves to perform the resistance training even if they had not experienced a change in vasomotor symptoms. Likewise, self-discipline and stubbornness were regarded as important personality traits to adhere to the resistance training. This was in part related to hopes of symptom relief since they did not experience an immediate effect on their symptoms. They knew that they could not expect an effect if they did not do the training and they convinced themselves to exercise in the hope to experience effects further on.

*Well, now it is to keep in shape. I mean I have been in a bad state in both body and, not the soul, but physically I have been in a very bad shape, and I feel that I become stronger when I exercise, and I need that. I may not get a lot of aerobic fitness, because I do mostly strength training, but I still feel that my body improves. It does. But I still can’t say that I think it’s fun to go and exercise, but I go and do what I am supposed to and then I leave. But I do it. I am stubborn in that way (Participant 9)*

To succeed in incorporating regular resistance training into their lives, participants had to adjust their life schedule and priorities. Many adjusted their schedule at work to be able to exercise before work in the morning or left earlier in the afternoon. The women planned and scheduled the exercise which was especially important for those living with a partner and/or children. It could be challenging when they had to take the needs of others into account, and for those who had little control over their work schedule. Those without stay-at-home children highlighted this fact as facilitating, and some saw the exercise to focus on themselves instead of others.

To have to travel a long way to work or to the gym made it more challenging to accomplish the regular resistance training; it was then mostly performed in the evenings or weekends. Most participants had to give up other activities to fit exercise into their lives. For many, the whole family was affected and had to adapt to new routines. Notably, although they had to make many adjustments and change priorities, the participants did not consider it a problem. Most meant that there had been no barriers to perform the exercise, but at the same time revealed they had made big changes to their routines to be able to do it. Their motivation made it natural to make the necessary adjustments and changes in their lives.

*No, it wasn’t really a barrier, but it was just that I had to leave my workplace so to speak, but they were nice and we tried to make it work as good as possible then, because she (the physiotherapist) couldn’t, I couldn’t go and exercise in the morning because I worked at an outpatient clinic, and she had the workout class at six p.m. so I had to go during my working hours, a part of them so to speak, so that could be one, but apart from that it was just to get in the car and go, so it wasn’t harder than that (Participant 11)*

To feed the motivation during the intervention, there was a need for both practical and emotional support. The push and support from the physiotherapist were important for motivation throughout the intervention. The participants trained harder during the sessions with the physiotherapist as they were pushed beyond their own limits of expectation. The physiotherapist encouraged them to continue when they became tired during the sessions, and not stop at a comfortable level of exhaustion. The physiotherapist also made them increase the resistance on the machines more than they had dared to do on their own. They trusted the physiotherapist to judge the limit of their capability and followed the directions they received.

*…the body protests, that is interesting too, like the lactic acid causes it to just stop, there is no power left (…) I have tried to increase the weights myself or do more repetitions (…) and I believe, well I have cleared that I can do it, but you need someone to push it you know, I can imagine it is, could be, it is tempting to stop when you start to get tired and not continue anyway until you just can’t do it anymore. And it is that limit that she (the physiotherapist) sees. You can do some more, I see it, you can do it. (Participant 1)*

To receive feedback on their technique and performance from the physiotherapist gave the participants a sense of security and encouraged them to continue when they knew they were doing it right. During the intervention, the participants felt increasingly competent and secure in the gym. They became comfortable with the program, the exercises, and the machines, and felt more at home in the gym environment. As they felt more competent, they needed less instructions and “push” from the physiotherapist.

*Otherwise, I wouldn’t have dared to join this resistance-training program, because I felt a bit like, curious but not eager enough to like, go in there by myself and put myself there. But now I’ve got it going, and now I think it is fun actually.* (I: What is the difference now…) *That someone has shown me how everything works. She has shown me how all the machines work and what exercises I should do and how I should perform the exercises. So, she kind of started something that I now have got with me. And that I now can, like, I dare to try new things now because she has shown me so well. (Participant 7)*

The environment at the gym also contributed to a positive experience for the participants during the training sessions; a place they liked to return to. They felt welcome there and found the gym staff friendly and helpful. The women appreciated the mixture of ages, gender, and fitness levels at the gym, resulting in a sense that “everyone was welcome”. To see other people like themselves do resistance training was motivating, and they appreciated to see people exercise with whom they could identify. They appreciated that the gym was clean with plenty of space most of the time, but they preferred to exercise outside rush hours to avoid the crowds.

*All my preconceptions about the gym have also disappeared. It is geeky and all that, but no, there were all kinds of people...all kinds that exercise, from young to old to rehab people and so on, it is amazing (Participant 6)*

After a while, participants started to recognize and befriend other people at the gym, and in that way the training sessions also became an opportunity of social encounter. Even though they exercised independently, they could interact with both staff and other people who exercised. However, some of the participants felt alone when they performed the training. For these participants, the weekly contact with the physiotherapist was even more important. They looked forward to seeing the physiotherapist every week, since it meant they would meet someone they knew. To meet the physiotherapist gave them a feeling of being important and validated. Some participants involved their partner and/or children and went to the gym together. After the intervention, some women joined group exercise sessions instead.

*…these two last times there has been a young, new mother, who has had her little baby with her… And we started to talk to each other… I mean, two from each her own perspective about being a woman and she thought it felt so good to start exercising again and she had had to adjust it a bit because of the circumstances and so on, so, well in 30-40 years it may still be relevant but for other reasons (Participant 1)*

#### Exercise to feel good

The women started noticing the physical effects of the resistance training some weeks into the intervention. They felt stronger, had more energy in every-day life, and experienced less muscle and joint pain. They were less limited by their physical ability and physically demanding activities had become easier. Several noted having a more muscular body composition and that clothes fit them differently. These changes were rewarding and sparked their motivation to exercise to maintain the physical effects. The resistance training made them feel better about their bodies and they wanted to keep that feeling. It was also motivating to be able to increase the loads in the resistance machines. When the women increased the loads, it was a clear and easy indicator that they had become stronger, and they described it as fun and inspiring to follow their progress over the weeks. Some were chasing higher loads and competing with themselves to reach the next level.

*Well, the motivation comes from increasing the loads regularly. That was the motivation. You had a bit in these machines, you knew that I could reach that, then I must increase so I reach the next. (Participant 8)*

*To feel good, yes in my age, and feel that the body can manage what it’s supposed to. That I’m not stiff, no pain or illness or, of course that can happen but it’s a way to keep my health and all. Yes, and it is nice to feel like, I manage to walk the stairs, I can lift that thing, it’s good for both body and mind. (Participant 12)*

The women felt proud to exercise, and at the end of the intervention they looked upon themselves as “someone who exercises”. They started to reshape their identity and consider themselves to be persons who exercise, instead of persons who used to exercise or were sedentary. They began to enjoy the training because it gave them a sense of well-being. Some noted that they had had negative preconceptions about resistance training before but changed during the intervention to consider it fun and enjoyable. To exercise generated feelings of happiness, and some meant they had started to crave and long for exercise.

*And you become proud, like, I exercise. Oh, how often. Yeah well, three times per week. It’s fun to be able to say that. (Participant 3)*

*You see that you become stronger, and you become stronger, and you can really, you feel physically strong. And it’s cool to get to feel so much better. Because of course, you get a kick from working out, you do. But you must experience, get there to get the effect. It’s like when I run, it’s equally boring the first 15 minutes, but then the effect comes, then these endorphins come, and that’s what you’re after. It feels like a high. And you want that because it makes you feel good-. (Participant 11)*

Although the trigger to apply for the intervention trial was the hope to relieve menopausal symptoms, all participants planned to continue to exercise regularly after the intervention had ended regardless of if they had had an effect on vasomotor symptoms. Some continued to perform the same resistance training program as during the intervention, while others had started to run regularly, attended group exercise classes, or did resistance training but followed a different program. Some participants had changed their dietary habits to eat more “healthy food” and decreased their sugar intake. In some cases, their partner and family had changed habits too.

*Yes, I think a bit more about what I eat, so now I have joined one of these where you count calories and try to achieve, to lose weight and, so I have changed that. I work out now, I didn’t do that before. I mean, I haven’t done that in ten years probably (…) Because my teenager said, I would never have believed that mum and dad would sit and plan like, on that day I will exercise, on that day you will exercise, you will have to pick up (the kids) and I will drop them off. Yes, all of us have changed, become affected. (Participant 13)*

### One year follow-up—finding new triggers

At the 1-year follow up most participants did no longer perform resistance training as in the original 15-week intervention. A few still followed the same program and almost half of the women did muscle-strengthening activity. However, almost all women exercised regularly, although some did only low-/or moderate-intensity physical activity like daily walks. Others performed only aerobic exercise or mixed modalities like circuit training with both aerobic and muscle-strengthening elements. Almost all described that they had changed their lifestyle compared to study-start with new habits of regular intentional physical activity.

Some women had experienced more climacteric symptoms when they reduced their resistance training and were once again triggered by hopes of symptom relief to increase the resistance training. However, the main motivators for performing resistance training or another type of exercise were the feelings of well-being that it gave—the sense of being strong and feeling good, and to do something positive for their health. Some women had stopped resistance training but thought about starting again because they missed the feeling that the resistance training gave them. They meant they had the “recipe” for success now since they knew they had succeeded before and therefore could do it again.

Establishing a routine was found to be an important factor to continue regular resistance training or other exercise. For those who had a pause or completely stopped resistance training, one barrier to continue was a life event that interrupted previous routines. It was hard to start training again after a break. Illness, bodily pain or a health condition that limited resistance training, even for a short period, were factors that affected. Moving to another area with a longer distance to the gym was a barrier to keep up the training, as well as having changed work hours or responsibilities. It was found that those who had kept up with the resistance training had established a routine—they were doing it without thinking.

The data from the 1-year follow up confirmed the finding in the analysis of a motivation that changed during the trial but still fuelled the exercise habits. The motivation was no longer linked to the symptoms; instead, the women had found their own motivation.

## Discussion

This study explored previously low-active postmenopausal women’s experience of participating in a resistance-training intervention for VMS and further aimed to find barriers and motivators for the training. The analysis generated three themes that were involved at different time points. These were: “Trigger—Hopes of symptom relief”, “An evolving motivation as a driving force for change” and “Finding new triggers”*.* The theme “An evolving motivation…*”* included a progression through *accountability, need for support* and *exercise to feel good.* Although the intention of the original intervention was not primarily to affect exercise habits but to treat VMS, all women had increased their physical activity at the 1-year follow-up telephone interview compared to baseline. About half of participants performed resistance training regularly 1 year after the intervention had ended.

There was a shared trigger for the women who entered the study, i.e., they all mainly wished to get relief from VMS and were willing to try exercise as a method. Although some had exercised regularly in the past, they had low physical activity levels at inclusion, and no one performed regular resistance training. Their main priority was not to start exercising per se, but to improve their menopausal symptoms and they were willing to act for that to happen. In that sense, the start-out point in our trial is different from intervention trials where increased exercise is the goal and participants often referred to exercise by a health professional [20]. The women in this study had all applied with the knowledge it was an exercise trial and thus already begun to take action to improve their symptoms. This is similar to findings from a study by Viljoen et al. who studied motivating factors with a quantitative method in peri- and postmenopausal women during and after a 24-month resistance training intervention to counteract cardiovascular risk factors [21]. They found a wish to improve health problems and the need for a structured exercise program acted as major motivators for the initial contact and early phase of the trial, while adherence during the trial was influenced by social contact with other participants and obligation towards the researcher [21].

Our analysis similarly found an evolving motivation that influenced and drove the resistance training in the women, from being triggered by the need for alleviation from distressing symptoms to something they did for enjoyment and general wellbeing. Early during the intervention feelings of obligation acted as motivators, as the women felt accountable to the researchers and the physiotherapist. Previous studies have described obligation as introjected regulation, important for early adoption of a physical activity program [21, 22]. During the course of the intervention study it was motivating to increase the loads in the resistance machines regularly and thereby see measurable effects from the training. The women in our study also felt accountable towards themselves to follow through with the exercise. Several described the resistance training as a chance to focus on themselves instead of others, and they did not want to let themselves down. In previous studies, the multiple roles and responsibilities from work, household, childcare, and partnership were barriers to exercise among younger and mid-aged women, as well as prioritizing the needs of others [23–25]. In contrast, almost all women in this trial denied they had experienced any barriers to resistance training during the intervention, although they described that they had to adapt their everyday lives to be able to exercise. Because of their motivation to follow through with the exercise intervention, they did the necessary adaptations and did not experience them as barriers. This finding was unexpected since partaking in the intervention meant a significant lifestyle change for the women who were all previously low-active. It was probably a combination of the strong motivation to participate, and the support they received from partners/family and the study’s physiotherapist, that contributed to the experience of having no barriers. The women used strategies that have previously been found to be successful, such as scheduling the training, and setting goals for improvement [26].

It has been described that support, from significant others or professionals, is important especially in the beginning of an exercise intervention [22, 27]. Accordingly, the women in our trial expressed the importance of both emotional and more “hands-on” kind of support. The guidance from the physiotherapist during the intervention was crucial because most women had no experience of resistance training. The support was important for practical reasons, e.g. to receive a structured program, assurance that they did the exercises correctly, the push to increase loads. In addition, the emotional support, and the fact that someone kept track of them was important. Kinnafick and colleagues found that those who embraced the available support in the beginning of a physical activity intervention were more likely to adhere to physical activity at follow-up after 6 and 10 months although they no longer needed the same amount of support. Likewise, a lack of support has been found to be a barrier to physical activity and exercise among mid-aged and older postmenopausal women [28]. In this trial, about half of the women did resistance training at the 1-year follow up and all did regular exercise or physical activity of some kind. It is possible that the support experienced by the women in our trial during the intervention contributed to them continuing to exercise at follow-up.

Interestingly, the effect on VMS was only partly important to the women to motivate them to exercise during the intervention. Published results from the intervention trial showed that VMS in the intervention group were almost halved, and that quality-of-life scores improved in several domains. It could have been expected that a positive treatment effect would act as positive feedback and be a trigger to continue the training. On the contrary, our analysis found that the motivation to exercise during the trial continued to evolve in all women regardless of their experience of treatment effect. Both women who noticed a treatment effect and those who did not, instead described how resistance training made them feel good and increased general well-being regardless of change in VMS. It was evident that the motivation had evolved from being external with a wish to relieve symptoms, to more internal with the aim to find a sense of well-being. This is supported by earlier studies which found that a resistance training intervention can increase self-efficacy and internal motivation to exercise [29, 30].

How the motivation changed during the study may have affected the exercise- and physical activity habits at the 1-year follow-up. When the support from the intervention ended, the women had to manage on their own to continue exercise. In line with previous data, we found that those who still performed regular resistance training had established a routine while those who had stopped resistance training had experienced life events that interrupted their previous routines. When they were no longer motivated by accountability and no longer supported by the physiotherapist, they started to perceive barriers such as competing demands and prioritizing others rather than themselves [25]. The women were at this stage mostly motivated by general well-being and positive feelings from exercise and possibly they found that they could achieve that through other types of exercise or physical activity than resistance training. This could have contributed to a transition towards other types of physical activity that they considered more fun or more easily accessible.

## Strengths and limitations

A strength with this study was the fact that the women who participated in the intervention trial were consecutively recruited to this qualitative study, regardless of compliance with the resistance-training intervention and regardless of effect on vasomotor symptoms. The intention was to not only capture the experiences of “successful” participants, but to get a broader perspective and richer data. Indeed, none of the invited women declined participation in this study, and all participated in the 1-year follow-up. There was a variation in age and life situations from newly postmenopausal women with full-time jobs and stay-at home children to those who were retired and more than 10 years after menopause.

The coding and construction of candidate themes was performed independently by the researchers and compared and discussed to check for coherence in the interpretation of data. The individual coding enabled a diverse range of codes to build candidate themes from, in line with the reflexive approach to thematic analysis. The original data were re-read several times throughout the analysis process and at the end to ensure that the themes were coherent with the data. Notes and documentation from the steps of the analysis process were saved to establish an audit trail.

The sample size of 15 participants could be considered small, however in this qualitative study setting using reflexive thematic analysis it is a sufficient number, due to richness and information of data in the interviews [31]. Malterud et al. used the term *Information Power*—the more relevant information that is generated through data collection, the fewer participants are needed. For example, a smaller sample size is needed when the participants have characteristics highly specific for the study aim and where the quality of the interview dialogue is strong [17]. In this study, the interviews were rich in data directly related to the aim, and almost the entire data set was relevant and coded. Recruitment for this qualitative study stopped when all eligible women until the third round of inclusion in the original RCT had been interviewed in this study. We regarded that the collected data was rich and meaningful, the sample was varied and that no obvious new data were generated during the last interviews. We therefore found it reasonable to finish recruitment [17, 18].

Since this is a qualitative study, it is in its nature not generalizable to other populations, and care must be given to the local context when interpreting the findings. However, we find that it may offer an insight that cannot be obtained by analysis of quantitative data with predefined categories. We aimed to examine the experience of a resistance training intervention in postmenopausal women. Globally, women are less physically active than men and physical activity decreases with age. It is estimated that barely a fifth of women around menopause perform enough muscle-strengthening activities as recommended to preserve health [32, 33]. This study provides some insight that could be considered when planning exercise referral for postmenopausal women.

## Conclusion

This first qualitative evaluation of physical exercise as an intervention to treat VMS in postmenopausal women, found that VMS acted as a motivational trigger to initiate resistance training in previously low-active women. Accountability, and continuous professional and emotional support, were factors that fueled the women’s motivation to perform regular resistance training during the study. Resistance training improved general well-being and most women experienced improvement in VMS. Interestingly, although the women initially were motivated by the hopes to be relieved from VMS, the motivation to exercise changed during the intervention into something the women did for enjoyment and well-being in general. This evolving motivation may have contributed to a long-term behavior change since all participants had increased their physical activity after 1 year regardless of effects on VMS.

## Supplementary Information

**Additional file 1.** Inclusion and exclusion criteria in the original RCT that investigated the effect of resistance training on menopausal hot flushes.

## Data Availability

The data set (transcripts in Swedish) generated and analysed during the current study is available from the corresponding author upon request from the corresponding author. Due to ethical reasons, to protect the integrity of participants, it is not publicly available.

## Abbreviations

MHT: Menopausal hormone therapy
VMS: Vasomotor symptoms
