# Supplementary material for: Postmenopausal women's experiences of a resistance training intervention against vasomotor symptoms: a qualitative study
Source: BMC Womens Health. 2022 Jul 30;22:320. doi: 10.1186/s12905-022-01900-0 (PMC9338607; doi:10.1186/s12905-022-01900-0)
Supplement: Supplementary file 1 — Additional file 1. Inclusion and exclusion criteria in the original RCT that investigated the effect of resistance training on menopausal hot flushes. [file 12905_2022_1900_MOESM1_ESM.docx]

| Inclusion criteria | Exclusion criteria |
| --- | --- |
| - Postmenopausal woman, defined as   - ≥ 12 months amenorrhea, or   - Hysterectomy and FSH >20 mIU/ml   - Amenorrhea due to intrauterine device including progestogen and FSH >20 mIU/ml   - Induced menopause after bilateral oophorectomy   - Induced menopause by chemotherapy or radiation and amenorrhea for at least two years     - - ≥4/day or 28/week moderate to severe hot flushes during a screening period of 2 weeks       - ≥45 years       - Good general health and physical ability to participate in resistance training for 60 minutes 3 days per week       - Able to understand and communicate in Swedish orally and in writing | - >75 minutes per week of moderate- to vigorous-intensity physical activity.^a^ - >225 minutes per week of physical activity of any intensity - Use of systemic menopausal hormone therapy during the last 2 months. - Unstable dose of selective serotonin and/or noradrenalin reuptake inhibitors or other substances, including natural remedies, with a possible effect on vasomotor symptoms - Any medical condition limiting the ability to participate fully in RT - Medical condition under investigation or condition that could affect hot flushes - Capillary hemoglobin <110 g/l - Systolic blood pressure >160 mmHg or diastolic blood pressure >100 mmHg |
| ^a^*Moderate intensity* = requires a moderate amount of effort and increases heart rate noticeably, like brisk walking. *Vigorous intensity* = requires a large amount of effort and increases heart and respiratory rate substantially, like running. | |

**Additional file 1. Inclusion and exclusion criteria in the original RCT that investigated the effect of resistance training on menopausal hot flushes.**
